# Polydopamine-Supported Lipid Bilayers

**DOI:** 10.3390/ma5122621

**Published:** 2012-12-04

**Authors:** Souryvanh Nirasay, Antonella Badia, Grégoire Leclair, Jerome P. Claverie, Isabelle Marcotte

**Affiliations:** 1 Department of Chemistry, Université du Québec à Montréal, C.P. 8888, Succursale Centre-Ville, Montréal, Québec H3C 3P8, Canada; E-Mail: nirasay.souryvanh@courrier.uqam.ca; 2 Department of Chemistry, Université de Montréal, C.P. 6128, Succursale Centre-Ville, Montréal, Québec H3C 3J7, Canada; E-Mail: antonella.badia@umontreal.ca; 3 Faculty of Pharmacy, Université de Montréal, C.P. 6128, Succursale Centre-Ville, Montréal, Québec H3C 3J7, Canada; E-Mail: gregoire.leclair@umontreal.ca

**Keywords:** supported membrane, phospholipid bilayer, polymer cushion, polydopamine, lipid diffusion

## Abstract

We report the formation of lipid membranes supported by a soft polymeric cushion of polydopamine. First, 20 nm thick polydopamine films were formed on mica substrates. Atomic force microscopy imaging indicated that these films were also soft with a surface roughness of 2 nm under hydrated conditions. A zwitterionic phospholipid bilayer was then deposited on the polydopamine cushion by fusion of dimyristoylphosphatidylcholine (DMPC) and dioleoylphosphatidylcholine (DOPC) vesicles. Polydopamine films preserved the lateral mobility of the phospholipids as shown by fluorescence microscopy recovery after photobleaching (FRAP) experiments. Diffusion coefficients of ~5.9 and 7.2 µm^2^ s^−1^ were respectively determined for DMPC and DOPC at room temperature, values which are characteristic of lipids in a free standing bilayer system.

## 1. Introduction

First reported in the early 1980s by McConnell *et al.* [1,2], supported lipid bilayers (SLBs) are commonly used as versatile biological membrane mimics. These two-dimensional soft systems are choice models to study the structure and function of the cellular membrane and its components. For example, they allow the investigation of lipid-lipid and cell-cell interactions, cell fusion, the functional role of membrane proteins, membrane-protein interactions, as well as other biochemical processes such as molecular transport, signaling, and catalysis [3]. Solid-supported membranes or solid-supported lipid bilayers (s-SLBs) consist of a continuous lipid bilayer deposited onto a planar solid substrate. An ultrathin water layer separates the membrane from the solid surface by a distance of 10–20 Å [4]. Mica, glass, and silicon oxide are the most commonly used substrates for s-SLBs [5,6,7]. In fact, hydrophilic, smooth, and clean substrates with little or no defects over a large area (of the order of 1 cm^2^) are preferred to support a high quality membrane [4].

SLBs can be prepared with different lipid mixtures and characterized with a wide array of surface-sensitive characterization techniques including atomic force microscopy (AFM), fluorescence microscopy, surface plasmon resonance, and ellipsometry. The distance between the lower leaflet of the bilayer and the solid support is not sufficient to prevent lipid-solid surface interactions and ensuing frictions [8]. This leads to a decrease in lipid mobility and, often, denaturation of incorporated transmembrane proteins [8]. Therefore s-SLBs do not fully account for the natural fluidity of biological membranes. One popular approach to address this issue is to deposit the lipid bilayer on a soft hydrated polymeric cushion. This system, called “polymer-supported membrane” was developed in the 1990s by Sackmann *et al.* [9,10]. The polymeric cushion acts as a lubricating layer between the bilayer and the solid surface, thus preserving the lipid mobility and membrane fluidity. According to Sackmann and coworkers, the polymer cushion should be soft, hydrophilic, not too highly charged, and not extensively cross-linked [9] with a thickness less than 100 nm [8].

In this work, we describe the preparation of polymer-supported lipid membranes using polydopamine as a soft polymeric cushion. Polydopamine films were introduced a few years ago as multifunctional and versatile polymer coatings [11,12]. They have the interesting ability to adhere on to either hydrophilic or hydrophobic materials. Polydopamine-based coatings form an efficient platform for the elaboration of antibacterial materials [13,14], the grafting of biomolecules, protein immobilization in biosensing devices [15], and controlling cell adhesion [16]. Inspired by the chemical composition of mussel adhesive proteins, they are prepared in a one-step process via oxidative self-polymerization of dopamine when introduced in alkaline solution [11,12]. The film thickness can be easily controlled by the immersion time of the substrate in the dopamine solution.

The mechanism of polydopamine polymerization is not completely understood. Recent progress by Dreyer *et al.* [17] suggests that polydopamine is a supramolecular aggregate of monomers instead of a covalent polymer. The formation of polydopamine would involve three characteristic steps, *i.e.*, oxidation of phenolic hydroxyls to carbonyls, cyclization of the pendant amine and polymerization via charge transfer, and hydrogen bonding and/or π-stacking. Hong *et al.* [18], however, proposes that polydopamine is formed following two distinct pathways, *i.e.*, a non-covalent self-assembly of dopamine and its oxidative product leading to the formation of a supramolecular complex, whereas the second one is a covalent oxidative polymerization. While further efforts are needed to elucidate the mechanism of polydopamine formation, it remains a facile and versatile method to obtain coatings for biocompatible applications.

**Figure 1:**
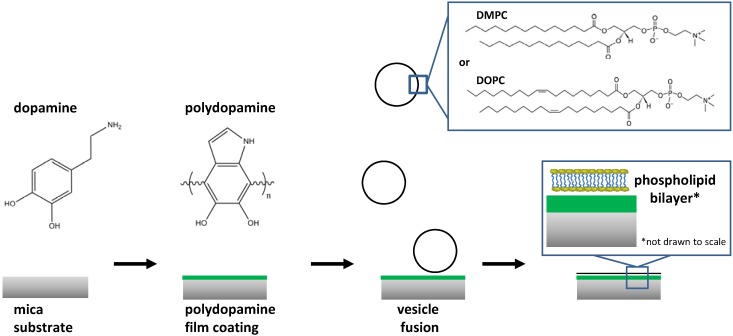
Preparation of polydopamine-supported membranes and the structure of polydopamine as suggested by Lee *et al.* [11].

In a previous report, we demonstrated that polydopamine was a suitable candidate for the preparation of polymer-supported membranes over nanoporous alumina filters containing pores of 73 to 200 nm in diameter [19]. The resulting assembly (nanoporous alumina surface + polydopamine cushion + supported lipid bilayer) was found to be useful to assess drug permeability. In short, we demonstrated that a continuous lipid bilayer was supported over polydopamine pillars separated by 73–200 nm holes. However, the integrity and fluidity of the lipid bilayer in this complex system was not examined. In this work, we have prepared polymer-supported membranes over a continuous polydopamine film and assessed their fluidity (Figure 1).

## 2. Results and Discussion

### 2.1. Polydopamine Film Thickness

**Figure 2:**
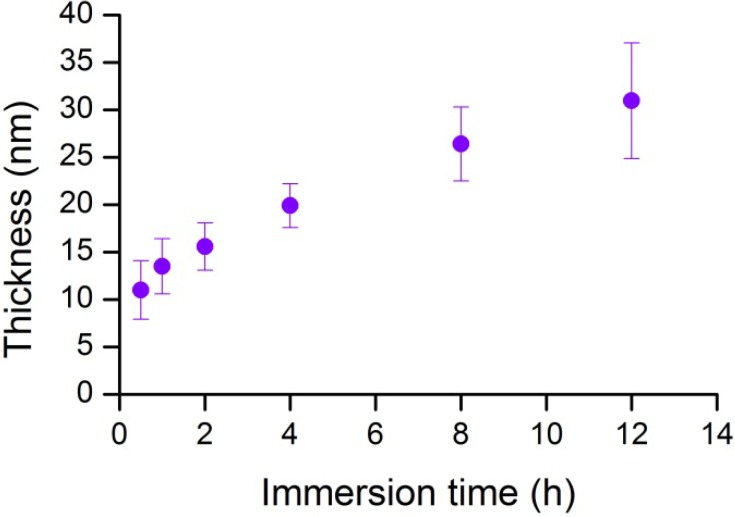
Effect of the immersion time (25 °C, phosphate buffer pH 8.5, dopamine = 2 g/L) on the polydopamine coating thickness as determined by ellipsometry in air.

In this study, we used mica as a solid support because its surface is smooth and flat at the atomic level after cleaving (in contrast to the nanoporous alumina used in our previous study [19]). The cleaved mica substrate was immersed in a dopamine solution of pH 8.5 (phosphate buffer) at room temperature, resulting in the formation of a polydopamine film (Figure 1). The polydopamine thickness as a function of immersion time was assessed by ellipsometry, as shown in Figure 2. Since this technique requires a reflective surface, the polydopamine coating was deposited on a substrate covered with a 50 nm aluminum layer obtained via thermal evaporation. For all other experiments, pure mica (no aluminum coating) was employed. A 20 nm thickness (dry film state) was determined after a 4 h immersion (Figure 2), in agreement with the literature [11]. The layer thickness did not increase linearly with immersion time and tends to level off after prolonged immersions (e.g., 33 nm for 12 h), suggesting that highly porous films are obtained at short immersion times which become denser with an increased immersion period. Previous studies showed that prolonging the immersion time to 24 h leads to a plateau and that the thickness gradually reaches a constant value [11,20]. In view of supporting lipid membranes, we selected an immersion time of 4 h as it yields a polydopamine coating thick enough to prevent the presence of uncoated spots on the substrate.

### 2.2. Mica and Polydopamine Film Characterization

**Figure 3:**
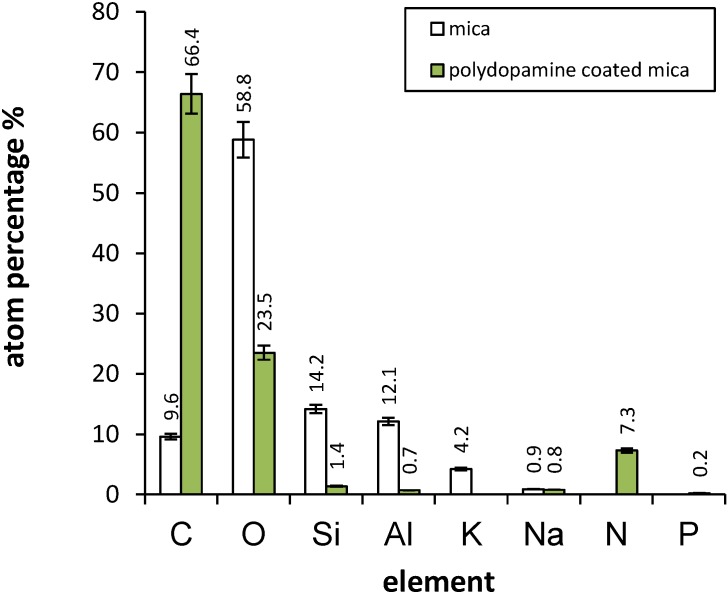
Surface atomic composition of mica and polydopamine-coated mica as determined by X-ray photoelectron spectroscopy (XPS).

The chemical composition of the mica surface and its modification by polydopamine were determined using X-ray photoelectron spectroscopy (XPS) analysis. This technique provides the atomic percentage of elements in the first 10 nm of the surface. Analyses were carried out on freshly cleaved mica and polydopamine-coated mica. Results are presented in Figure 3.

Percentage values obtained in the case of cleaved mica are similar to those from Shlyakhtenko *et al.* [21]. The presence of adventitious carbon as contaminant is expected. After polydopamine modification, we observe a significant increase in carbon as well as nitrogen concentrations, and a concomitant decrease in Si, Al and K content. This indicates the effective deposition of a polydopamine layer on the surface as C and N are polydopamine elements whereas Si, Al and K are mica constituents. Our XPS results are in agreement with those found by Li *et al.* [22] for a polydopamine layer. Calculation of the N/C percentage ratio for the polydopamine-coated mica reveals a value of 0.110 close to the theoretical value of 0.125 (1N for 8C) for pure dopamine. We also notice only traces of phosphorus originating from the phosphate buffer, probably due to insufficient rinsing, indicating that this element is not incorporated into the composition of polydopamine during the film formation.

**Figure 4:**
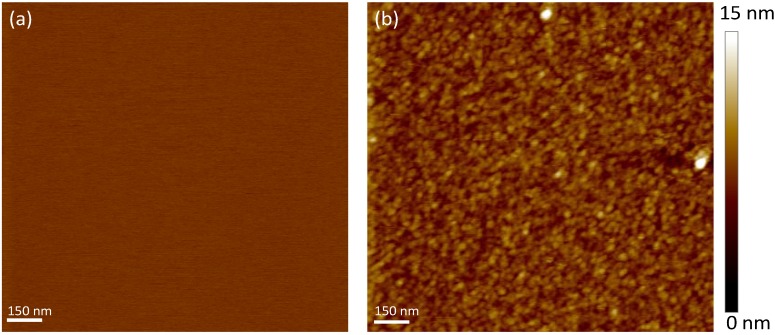
Atomic force microscopy (AFM) topography of (**a**) unmodified and (**b**) polydopamine-coated mica in water using tapping mode (scan size 1.5 µm × 1.5 µm). *Z*-scale is shown on the right side.

The polydopamine film was further characterized by atomic force microscopy (AFM) in tapping mode in deionized water. Images of the mica surface before and after polydopamine coating are shown in Figure 4. Unmodified mica has an atomically flat surface as indicated by the root mean squared (RMS) roughness value of the bare substrate of ~0.2 nm for an area of 5 × 5 µm^2^. After polydopamine film formation, a uniformly covered surface can be observed in addition to an increase in the surface roughness ~2.1 nm for an identical surface area. This value is small enough to consider the surface still flat [23]. Moreover, according to Richter *et al.* [24], roughness in the nanometer range has little effect on the bilayer formation. Interestingly, the film morphology reveals the presence of round and packed particles with a diameter of ~30 nm, suggesting that the polymer grows with a granular structure on the mica surface. Altogether, our XPS and AFM results show that a thin cushion of polydopamine can fully cover the mica surface.

To investigate the mechanical properties of the polydopamine film, AFM was used to obtain force distance curves (commonly called force curves). By indenting the AFM tip on the sample surface, the cantilever deflection is recorded as a function of the piezoelectric displacement *z*. Because the deflection is a measure of the force between the sample surface and the tip, the force value can be extracted, providing valuable information on the elastic properties of the sample. Indeed the slope of the curve allows calculating the elastic Young modulus E as described by Domke and Radmacher [25]. Combination of Hooke’s law (relation between the cantilever deflection and the applied force) with the Hertz model (relation between the indentation and the applied force) gives the following equation:

(1) $$z-z_{0}=d-d_{0}+\sqrt{\frac{k(d-d_{0})}{\frac{2}{\pi }\;[E\left(1-\nu^{2}\right)]\text{tan}(\alpha )}}$$

where *d*_0_ is the zero deflection, *z*_0_ is the contact point, ν is the Poisson ratio of the sample and α is the half opening angle of the tip (18° according to the manufacturer). The parameter *k* is the force constant of the cantilever and represents its stiffness. By taking two points on the slope of the curve, deflection values and their corresponding *z* values are used to deduce Young’s modulus E. To improve the accuracy of our measurements, tips were calibrated by determining the cantilever’s mechanical response to thermal noise. We found a *k* value of 0.046 N/m as compared to the manufacturer’s nominal value of ~0.03 N/m.

**Figure 5:**
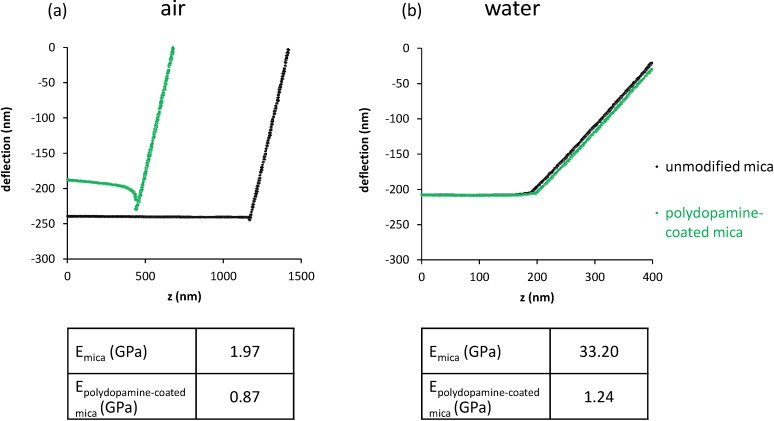
Force curves obtained (**a**) in air (**b**) in water for unmodified and polydopamine-coated mica, with corresponding calculated elastic moduli.

Figure 5 shows the results of the measurements carried out on dry as well as humid uncoated and polydopamine-coated mica. For the measurements corresponding to the plateau section of the curve, the tip is above the surface. Once the tip touches the surface, the deflection starts to increase. From the slope of the deflection as a function of the tip position, it is possible to assess the Young modulus of the material.

The small negative indentation for dry mica (also present to a smaller extent for polydopamine in air) is an expected phenomenon which is attributed to the presence of capillary and van der Waals forces when the tip is close to the surface [26]. A Young’s modulus of 1.97 GPa was calculated for dry mica whereas the value obtained in water was 33.20 GPa. The difference of Young modulus between mica in air and in water is expected as the contribution of van der Waals forces is different in both environments [26]. In the case of dry polydopamine-coated mica, a Young modulus of 0.87 GPa is found, *i.e.*, 2.2 times smaller than unmodified mica. In water, though, E is 27 times smaller (1.24 GPa) than that of unmodified mica. We can thus conclude that the polymer cushion is significantly softer than mica, most notably in water, where the polymer is probably hydrated and swollen by water.

### 2.3. Characterization of DMPC and DOPC Bilayers on Polydopamine-Coated Mica

Dimyristoylphosphatiylcholine (DMPC) and dioleoylPC (DOPC) bilayers were deposited on the polydopamine-coated mica using direct vesicle fusion. The choice of these lipids was motivated by the abundance of phosphatidylcholines in the eukaryote membrane composition and their widespread use in membrane mimetics [27]. In addition, their deposition on polymer supports has already been extensively scrutinized (see below, Table 1). The fusion is achieved by heating the system above the phase transition temperature of the phospholipids, which is 23 °C for DMPC and −20 °C for DOPC [28]. After fusion, samples were rinsed with deionized water to remove the excess of vesicles and they were never left to dry.

SLB formation by thermal vesicle fusion depends on several factors associated to the nature of both the polymer support and lipids which will affect the interaction between them. More specifically, the polymer surface charge, hydrophilicity, and roughness play an important role, as well as the lipid charge, size, and phase [29,30,31,32,33]. Other parameters such as the buffer composition, pH, and ionic strength need to be considered [24]. The formation of SLBs occur after adsorption of the vesicles onto the support and fusion of neighboring vesicles to form bigger ones, followed by their rupture into bilayer patches. Then adjacent bilayers patches coalesce and ideally grow until a complete supported phospholipid bilayer is obtained [24].

**Figure 6:**
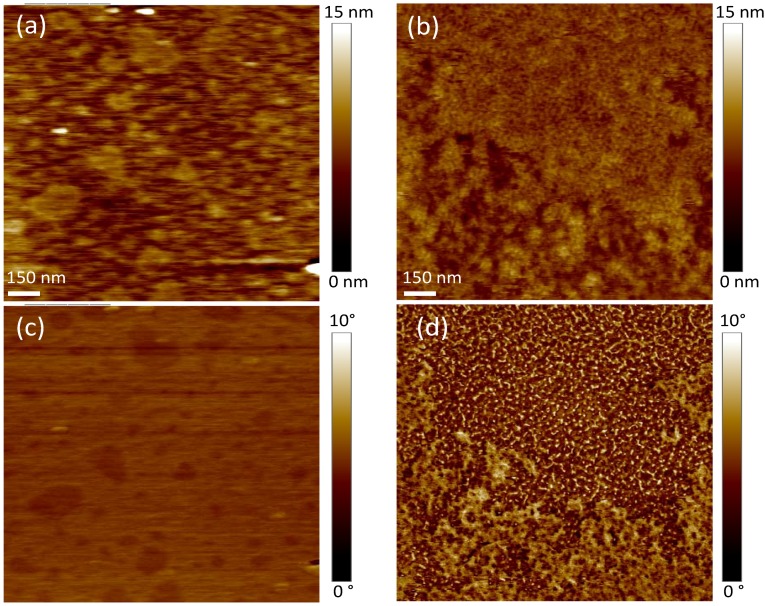
Tapping mode AFM images in deionized water of DMPC (left) or DOPC (right) supported on polydopamine-coated mica (top: height mode, bottom: phase mode, scan size 1.5 µm × 1.5 µm).

AFM imaging was performed to verify the deposition of the bilayers on the polydopamine cushion. As shown in the topography images presented in Figure 6a and Figure 6b, both phospholipids do not fully cover the polymer surface. Wagner and Tamm observed that lipid bilayers formed on polymers are often patchy and present structural defects [34]. Such bilayer spots of DMPC (Figure 6c) and DOPC (Figure 6d) are easier to visualize on phase images, contrasted in dark. AFM phase imaging is sensitive to the viscoelastic behavior and chemical heterogeneities of the material, and therefore, the phospholipid bilayer, which is softer than the polymer, can be clearly distinguished by this method. We also measured bilayer thicknesses of 3.5 ± 1.5 nm for DMPC and 4.0 ± 1.5 nm for DOPC. These values are comparable to other reported data for DMPC [35,36] and DOPC [37] bilayers formed on mica. In addition, RMS roughness values of ~1.3 nm for DMPC and ~1.7 nm for DOPC (area of 5 µm × 5 µm) were determined for the bilayers. These values are larger compared to those of DMPC and DOPC bilayers supported on mica (~0.4 nm, results not shown). However, they are slightly smaller than the RMS roughness of the polydopamine-coated surface ~2.1 nm, indicating the bilayers follow the topography of the polymer.

The adsorption of the vesicles onto the polydopamine cushion can first be explained by the use of a phosphate buffer which favors the contact of the vesicles with the polymer cushion surface, leading to bilayer formation for lipids containing PC head groups. Indeed at this ionic strength, the Debye length is very small (0.7 nm) [38] but the Na^+^ ions located in the electrical double layer of the surface are small enough to allow the approach of the vesicles to the surface [39]. When a vesicle comes near the surface, Na^+^ ions must remain in the vicinity of the negative charge (either on the surface or on the phospholipid) in order to maintain electroneutrality. Secondly, the interactions between the polydopamine cushion surface and DMPC or DOPC vesicles are sufficiently attractive to allow adsorption of the vesicles. DMPC and DOPC are zwitterionic phospholipids while the polydopamine film contains amine, catechol and quinone groups [15] and is negatively charged at physiological pH due to the deprotonation of one catechol (OH) group [40]. To our knowledge, the surface charge density of polydopamine has not been precisely measured but it can be estimated using space filling considerations [40]. 5,6-dihydroxy indole—the repeat unit of polydopamine (as indicated in Figure 1)—is essentially a flat molecule as modelled by molecular mechanics using MM2 as force field [41]. The Connolly molecular area calculated with this force field is 157 Å^2^, thus, with one face of the molecule exposed to the surface (the other side facing the bulk of the polymer), one deprotonated OH group occupies a surface of 157/2 = 78 Å^2^. This leads to an approximate charge density of 1.3 × 10^14^ negative charges per cm^2^ of polydopamine. Cha *et al.* [39] have recently demonstrated that phospholipid vesicles are adsorbed via attractive electrostatic interactions between the positive charge of the choline headgroups and negative charges of a surface, provided the surface charge density is larger than a critical surface charge density of 3 × 10^14^ negative charges per cm^2^. The presence of bilayer patches on the polydopamine cushion can most likely be ascribed to insufficient surface charge covering (1.3 × 10^14^ negative charges per cm^2^ *vs.* 3 × 10^14^ measured for a full covering).

**Figure 7:**
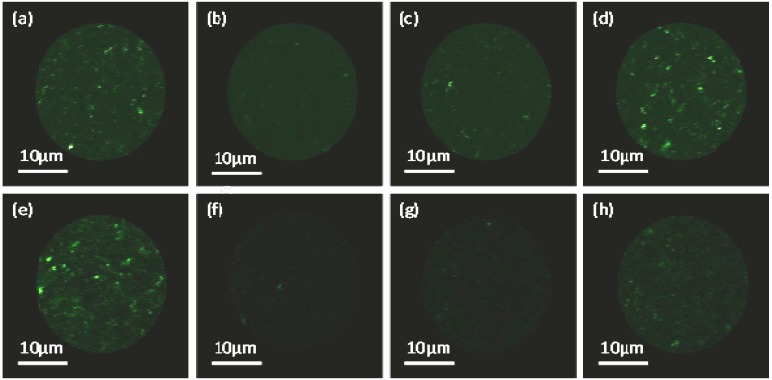
Fluorescence microscopy recovery after photobleaching (FRAP) images of phospholipid bilayers supported on polydopamine-coated mica: DMPC (**a**) before; (**b**) immediately after; (**c**) 3 s; and (**d**) 288 s after photobleaching. DOPC: (**e**) before; (**f**) immediately after; (**g**) 3 s; and (**h**) 201 s after photobleaching.

The mobility of phospholipids in the polydopamine supported bilayers was verified by fluorescence microscopy recovery after photobleaching (FRAP) experiments which allow calculation of the diffusion coefficient from the recovery of the fluorescence curve. Vesicles containing DMPC and DOPC with an Oregon green 1,2-dihexadecanoyl-sn-glycero-3-phosphoethanolamine (OG-DHPE) label were incubated with polydopamine-coated mica as previously described. Figure 7 shows the process of fluorescence recovery for DMPC (Figure 7a–d) and DOPC (Figure 7e–h) bilayers after bleaching a spot of 27 µm diameter. Almost complete recovery occurred after 5 min for DMPC and 4 min for DOPC bilayers.

**Figure 8:**
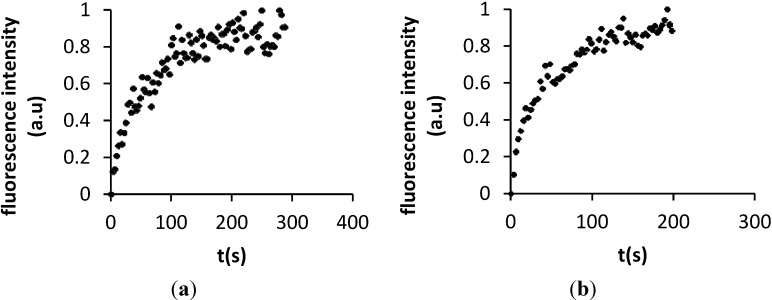
Determination of the mobility for (**a**) OG-DHPE labeled DMPC and (**b**) DOPC deposited onto polydopamine-coated mica from the FRAP normalised fluorescence intensity as a function of time.

Diffusion coefficients were calculated using the Soumpasis method (see Experimental Section) from the recovery curves displayed in Figure 8 [42]. Diffusion coefficients of 5.9 ± 2.1 µm^2^ s^−1^ and 7.2 ± 1.1 µm^2^ s^−1^ were respectively determined for DMPC and DOPC. This compares very well with the value of 7.8 µm^2^ s^−1^ reported for free standing DOPC bilayers [43]. In comparison, a diffusion coefficient of about 3.1 µm^2^ s^−1^ is reported on mica [43] while values of 2.16 to 3.20 µm^2^ s^−1^ have been found for polymer-cushioned membranes (see Table 1 which lists diffusion coefficients reported by other groups [30,44,45,46]).

The higher diffusion coefficients found for DMPC and DOPC bilayers supported on the polydopamine cushion as compared to unmodified mica indicate that the presence of the polymer coating enables the lateral mobility of the phospholipids. Moreover, the diffusion coefficients calculated for both lipids are characteristic of a fluid phase and similar to a free standing bilayer system. Our results also reveal that the diffusion coefficients achieved by DMPC and DOPC on polydopamine-coated mica surpass those reached for these lipids on other polymer cushions. We can thus conclude that the polydopamine coating fulfills its role of maintaining the membrane fluidity by reducing the frictional coupling between the bilayer and the mica surface.

**Table 1:** Lateral diffusion coefficients of lipids supported on various polymer supports, as measured by fluorescence recovery after photobleaching (FRAP).

Lipid	Polymer	Support	D (μm^2^/s)	Reference
DMPC	PAA	glass, quartz or silicon oxide	2.56 ± 0.84	[30]
DMPC/cholesterol/PEG-DMPE (1 mol %)	cellulose	glass	3.3 ± 0.2	[44]
DOPC	PEG	glass	2.16 ± 0.07	[45]
DOPC	(CHI/HA)_5_	glass	2.8 ± 0.2	[45]
PC/PE/PS/cholesterol	maleic acid	silicon oxide	0.26–2.6	[46]

PAA: poly(acrylic acid); PEG: (poly)ethylene glycol; CHI: chitosan; HA: hyaluronic acid.

## 3. Experimental Section

### 3.1. Materials

Ruby muscovite mica, ASTM V-1 quality, was obtained from B&M Mica Co. Inc. (Flushing, NY, USA). 1,2-dimyristoyl-sn-glycero-3-phosphocholine (DMPC) and 1,2-dioleoyl-sn-glycero-3-phosphocholine (DOPC) were purchased from Avanti Polar Lipids (Alabaster, AL, USA). Oregon green 488 1,2-dihexadecanoyl-sn-glycero-3-phosphoethanolamine (OG-DHPE) was bought from Life Technologies Inc. (Burlington, ON, Canada), while dopamine hydrochloride and all other chemicals were obtained from Sigma-Aldrich (Oakville, ON, Canada).

### 3.2. Coating of Mica with Polydopamine

A 2 g/L dopamine solution was prepared by dissolving 10 mg of dopamine hydrochloride powder in 5 mL of sodium phosphate buffer (100 mM, pH 8.5) prepared using nanopure water. The freshly cleaved mica substrate was immediately immersed in the solution and left for 4 h at room temperature. The mica substrate was then removed from the solution and extensively rinsed with deionized water, and finally dried under a stream of nitrogen.

### 3.3. Deposition of Phospholipid Bilayers

*For AFM imaging.* DMPC or DOPC were hydrated in sodium phosphate buffer (10 mM, pH 7.4) and submitted to a series of five freeze-thaw-vortex shaking cycle. The resulting multilamellar vesicles were then sonicated with a titanium sonicator probe (20 W, 15 s pulses for 20 min, each pulse separated by a 30 second dead time). They were then centrifuged at 5000 rpm for 30 min. The supernatant was extruded 30 times through 200 nm polycarbonate membranes at 40 °C for the DMPC suspension and at room temperature for DOPC. Freshly-prepared polydopamine-coated mica substrates were then immersed in the unilamellar vesicle suspensions and incubated overnight at 40 °C for DMPC bilayer formation and room temperature for DOPC. Finally, the samples were rinsed with deionized water to remove excess vesicles and these were never left to dry before imaging.

*For Fluorescence Microscopy and FRAP experiments.* Chloroform solutions of either DMPC/OG-DHPE or DOPC/OG-DHPE (molar ratio of 49:1) were first prepared with a total lipid concentration of 1 mM. Chloroform was then removed by rotary evaporation at 30 °C resulting in the formation of a dry lipid film on the inner surface of the round-bottom flask. An appropriate volume of sodium phosphate buffer (10 mM, pH 7.4) was added in each flask and films were allowed to hydrate for 1 h at 30 °C for the DMPC/OG-DHPE mixture and room temperature for DOPC/OG-DHPE. The resulting aqueous suspensions were also agitated and vortex shaken. This led to the obtention of multilamellar vesicles solutions. Unilamellar vesicles solutions were then obtained by bath-sonication (Branson B2510) for 10 min followed by extrusion as described above. The vesicle suspension was then deposited on polydopamine films using the procedure described above.

### 3.4. Ellipsometry

Thickness measurements of dry polydopamine films were carried out on monocrystalline silicon substrates coated with a 50 nm aluminum layer by thermal evaporation. Prior to metal surface deposition, silicon substrates were treated with H_2_SO_4_/H_2_O_2_, heavily rinsed with deionized water, and dried under a stream of nitrogen. The aluminum-coated silicon substrates were then immersed in a freshly prepared dopamine solution (as described above) for different times at room temperature. At the end of the incubation period, the samples were removed from the polydopamine solution, extensively rinsed with deionized water and dried with a gentle stream of nitrogen. The ellipsometry measurements were performed in air with a M-2000 spectroscopic ellipsometer (Woollam, Lincoln, NE, USA) with a wavelength scan from 370 to 1000 nm and at an angle of incidence of 75°. The polydopamine layer thickness was determined by fitting the plots of ψ and Δ *vs.* wavelength to a four-layer Si/SiO*_x_*/Al/polydopamine model using the Levenberg-Marquardt non-linear optimization algorithm of the vendor’s WVASE32^®^. The polydopamine layer was modeled as a Cauchy layer using the dispersion equation: *n*(*λ*/µm) = *A* + *B*/*λ*^2^ +*C*/*λ*^4^. *A* = 1.45, *B* = 0.01, and *C* = 0 were used to fit the data [20]. The results were repeated using three replicate samples (*N* = 3) and three measurements were performed on each sample.

### 3.5. X-Ray Photoelectron Spectroscopy

XPS experiments were performed with a PHI 5600-ci spectrophotometer (Physical Electronics, Eden Prairie, MN, USA). A standard Mg anode operated at 300 W was used for survey scans. Spectra were obtained with an electron take-off angle of 45° relative to the surface sample and a 0.8 mm area was analyzed. A charge neutralizer was used to avoid charging effect. Survey scans were obtained using pass energies between 0 and 1200 eV with a duration of 8 min for acquisition. XPS analyses were carried out on freshly cleaved/made mica and polydopamine-coated mica. The experiments were repeated with three different samples (*N* = 3).

### 3.6. Atomic Force Microscopy (AFM)

*Topography imaging.* Atomic force microscopy (AFM) pictures were taken in water with a Veeco Dimension 5000 microscope equipped with a Nanoscope V controller (Bruker/Veeco, Santa Barbara, CA, USA). Gold coated silicon nitride tips purchased from Bruker with a nominal spring constant of ~0.1 N/m and resonance frequencies between 8 and 25 kHz were used. Data analysis was performed using the NanoScope Analysis software (Version 1.30). All images were taken at room temperature using tapping mode.

*Force distance curves.* Force distance curves were obtained with a Veeco Dimension 5000 microscope equipped with a Nanoscope V controller. Gold-coated silicon nitride tips from Veeco with a nominal spring constant of ~0.03 N/m were used. To improve the accuracy of the measurements, tips were calibrated by measuring the cantilever’s mechanical response to thermal noise. Studies were performed in contact mode at 23 °C. Data analysis was performed using the NanoScope Analysis software (Version 1.30).

### 3.7. Fluorescence Microscopy and Fluorescence Recovery after Photobleaching (FRAP)

Fluorescence images were taken using a Nikon Ti A1R confocal laser scanning microscope equipped with a 100X/1.45 NA Plan Apo TIRF objective. Microscope examination was done using 488 and 515 ± 30 nm as excitation and emission wavelengths, respectively. FRAP experiments were performed by bleaching a 27 μm diameter spot during 8 s with full power laser using a 488 nm excitation wavelength. The FRAP was recorded at 3 s interval between images at a reduced power laser (3%). Data were analyzed using ImageJ software and diffusion coefficients determined using the Soumpasis method [42]. The diffusion coefficient was calculated using the following equation:

(2) $$D=0.224\;\frac{\omega^{2}}{t_{1/2}}$$

where *D* is the diffusion coefficient, *ω* is the radius of the photobleached spot and *t*_1/2_ the time at which half of the intensity was recovered. The experiment was repeated three times.

## 4. Conclusions

We have demonstrated that zwitterionic phospholipid bilayers can be supported on polydopamine-coated mica. Characterization of the modified mica surface by XPS, ellipsometry, and AFM confirmed full coating by a 20 nm soft polydopamine cushion with little surface roughness. The phospholipid bilayer was deposited by vesicle fusion and shown to follow the cushion topology. The capacity of the polydopamine cushion to preserve the phospholipid mobility was demonstrated by the diffusion coefficient of the phospholipids measured by FRAP, which are similar to values found for fluid-phase lipids in liposomes. Polydopamine biofilms are extremely easy to prepare and strongly adhere to a very large number of substrates without the need for covalent modification of the surface. Coupled to the fact that polydopamine cushions preserve lateral phospholipid mobility in the bilayer, the aforementioned advantages all point to polydopamine as an excellent choice to prepare polymer-supported membranes.
